# Experimental study on the evaporation of typical oil spills in the Bohai Sea

**DOI:** 10.1039/d6ra06144c

**Published:** 2026-09-18

**Authors:** Lan Wang, Xue-Ji Gu, Kai Su, Hai-Zhou Zhang, Chuan-Ting Zhao, Liang Zhang

**Affiliations:** a North China Sea Marine Forecasting, Hazard Mitigation Center, Ministry of Natural Resources Qingdao Shandong Province 266061 China xuejigu@hotmail.com; b Key Laboratory of Coastal Science and Integrated Management, Ministry of Natural Resources Qingdao Shandong Province 266061 China

## Abstract

With the rapid expansion of offshore oil exploration, production, and shipping, oil spill accidents have become increasingly frequent and pose growing threats to the marine environment. This study investigates the physicochemical properties and evaporation kinetics of five typical oil spill samples from the Bohai Sea (northern China, East Asia). The oils (three crude oils and two fuel oils) differ markedly in composition (SARA fractions and distillation cuts). Density, viscosity, and composition were characterized, and empirical models linking composition, temperature, and viscosity were developed. Evaporation experiments were conducted in a shallow-plate system (0.5 mm oil film) under controlled temperatures (7–40 °C) and wind speeds (0–6 m s^−1^) for eight days. Evaporation rate and maximum loss increased significantly with temperature and wind speed, following a strong logarithmic relationship with time (*r*^2^ > 0.95). Kinetic parameters were correlated with oil composition to establish a multi-factor empirical prediction equation. Changes in density and viscosity during evaporation were quantified, providing a basis for predicting oil-spill behavior in the Bohai Sea.

## Abstract 2

With the rapid expansion of offshore oil exploration, production, and shipping, oil spill accidents have become increasingly frequent and pose growing threats to the marine environment.

## Graphical abstract

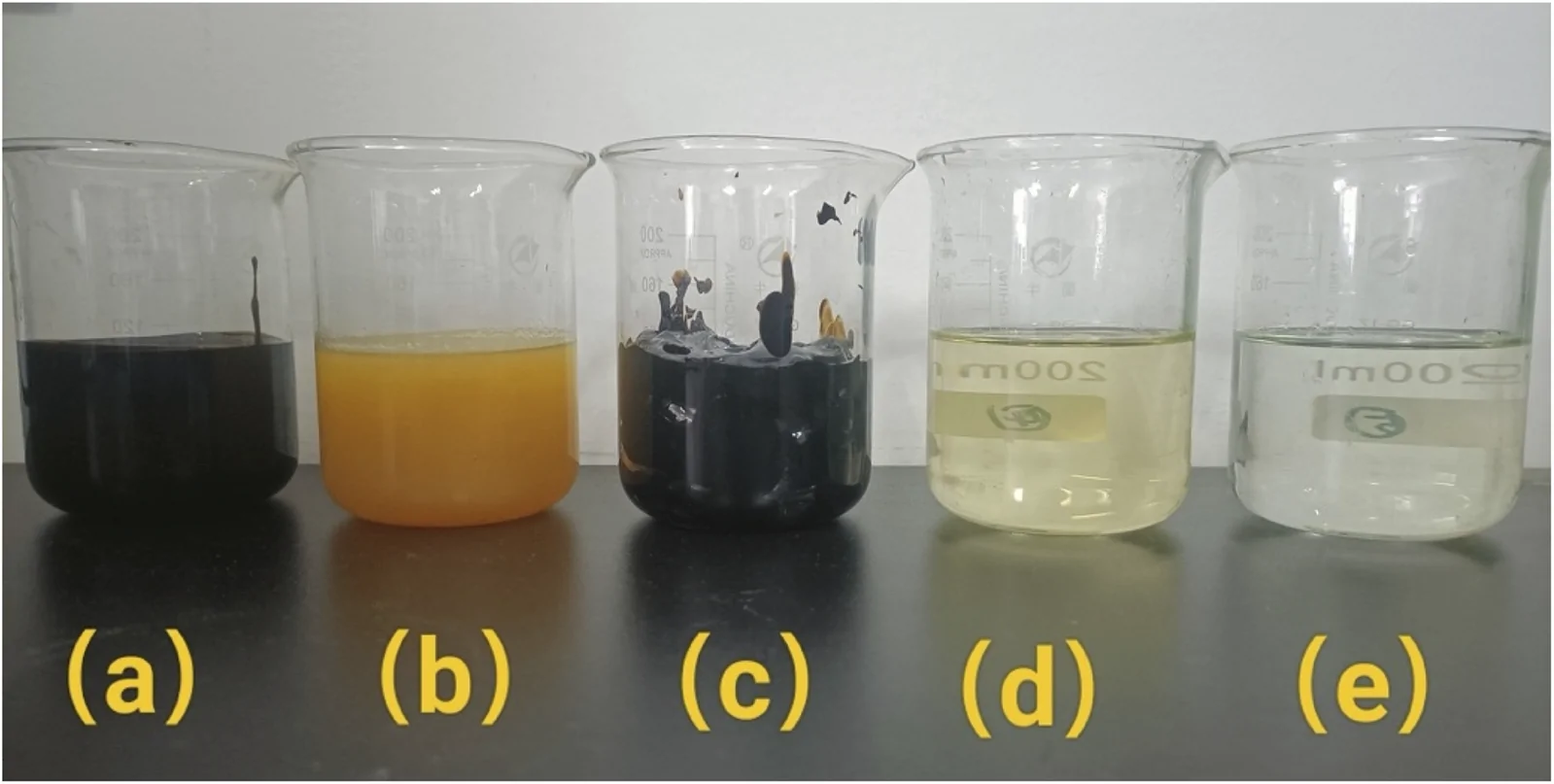

## 1 Introduction

Offshore oil exploration, production, and shipping have expanded rapidly in recent decades, leading to increasingly frequent oil spill accidents that pose serious threats to the marine ecological environment.^1,2^ The migration and transformation of petroleum in the marine environment constitutes a complex multiphase process, which is influenced by various factors, primarily environmental conditions, such as wind, waves, temperature, and ocean currents, and intrinsic oil properties, including viscosity, density, and composition.^3,4^ Owing to the complex mechanisms governing oil spill behavior, it is difficult to track and monitor its transformation process, which hinders accurate tracing and prediction of oil spills.^5,6^ Therefore, to minimize the risks of marine oil spill pollution and to develop and select effective response strategies, it is necessary to conduct in-depth studies on the dynamic evolution of oil spills and to simulate the oil weathering process.^7,8^ By quantitatively analyzing the mechanisms of oil weathering and the associated changes in physicochemical properties, the migration and transformation patterns under different environmental conditions can be evaluated.^9,10^

Typical crude oil can lose up to 45% of its initial volume through evaporation within several days.^11^ In the early stages of a spill, light crude oil can exhibit mass losses of up to 75%, and medium crude oil of approximately 40%, whereas heavy or residual oil generally loses only approximately 10%.^12^ Because the fate of oil spills in the ocean primarily depends on their physicochemical properties and environmental conditions,^13,14^ factors such as temperature and wind speed can significantly affect the evaporation rate and extent, consequently altering the fundamental characteristics of the spill.^15^ Therefore, an in-depth study of the evaporation process is crucial for accurately predicting the environmental behavior of oil spills in the ocean and for formulating effective emergency response and remediation strategies.^16,17^

Previous studies have relied on static experiments conducted under specific environmental conditions, which fail to capture the dynamic evaporation process under the coupling action of multiple factors in real marine environments.^18^ In addition, existing models still exhibit considerable uncertainty in predicting the temporal variation of physicochemical parameters for different oils.^5,19^ To address this issue, this laboratory-scale study selected five typical crude oils and fuel oils from the Bohai Sea to systematically investigate evaporation behavior. Rather than testing micro-scale colloidal suspensions, the experiments utilized a standardized shallow-plate evaporation system to simulate continuous thin oil slicks on the sea surface, with a fixed film thickness of 0.5 mm (utilizing 10–20 mL per dish depending on oil density). Environmental conditions were precisely regulated in a constant-temperature chamber (7–40 °C) equipped with an adjustable wind-generation device (0–6 m s^−1^) to isolate individual and coupled environmental effects. While laboratory-scale experiments provide high precision for determining intrinsic kinetics, scaling these empirical formulas to open-ocean spills presents challenges due to variable wave agitation, dynamic ocean currents, sunlight exposure, and multi-process weathering coupling (*e.g.*, photo-oxidation and emulsification). Nevertheless, these controlled laboratory measurements yield critical benchmark parameters for operational oil spill prediction models.

## 2 Materials and methods

### 2.1 Acquisition of experimental oils

This study focused on the Bohai Sea region and selected five typical crude and fuel oils as experimental samples, particularly three types of crude oil and two types of fuel oil: crude oils included Penglai 19-3 crude oil (PL), Bozhong 19-6 crude oil (BZ), and Caofeidian 6-4 crude oil (CFD); fuel oils included marine fuel oil (CY) and No.10 diesel (SH). Among these, PL, BZ, and CFD crude oils were collected from representative oilfields in the Bohai Sea. These oils cover a broad range of physicochemical properties: BZ is a light crude/condensate oil with lower density and viscosity; PL and CFD are medium-to-heavy crude oils enriched in resins and asphaltenes; CY is a heavy residual fuel oil; and SH is a light refined petroleum distillate. To establish a full material profile for these specific batches, all fundamental physical and chemical properties—including initial density, kinematic viscosity, SARA four-group fractions, distillation fractions, and *n*-alkane (C10–C38) distributions—were systematically measured in this work, with full numerical datasets detailed in Section 3.1 (Table 2 and Fig. 2).

After sampling, the oil samples were immediately transferred into clean, dedicated storage tanks, sealed, and labeled with unique identifiers. Subsequently, they were stored under dark, ventilated, and constant-temperature laboratory conditions until subsequent analysis and testing to ensure that the physicochemical properties of the samples remained stable during storage and avoid experimental data deviation caused by environmental factors. The initial oil samples are shown in Fig. 1.

**Fig. 1:**
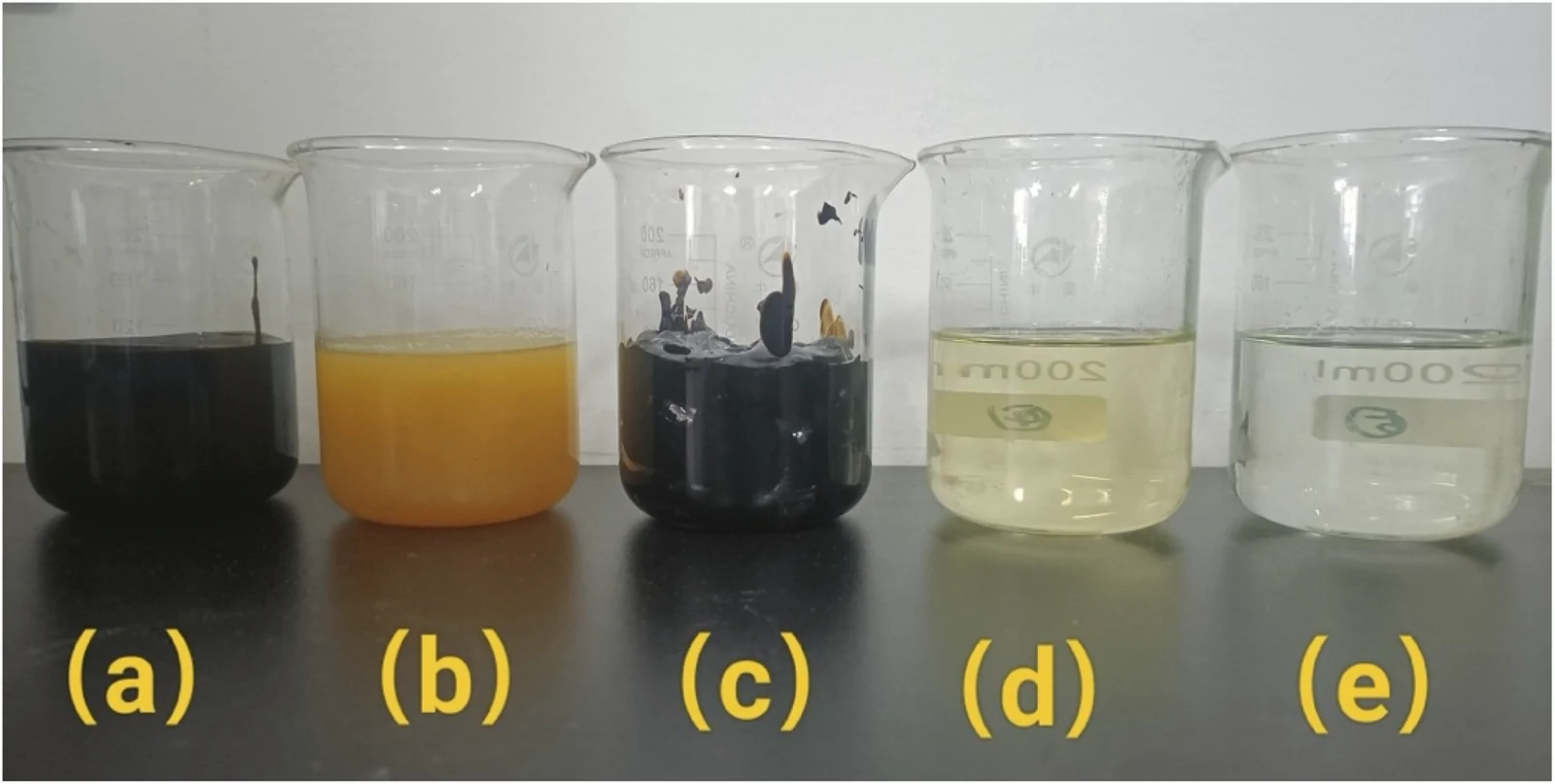
Experimental oil samples: (a) Penglai 19-3, (b) Bozhong 19-6, (c) Caofeidian 6-4, (d) marine fuel oil, and (e) No.10 diesel.

### 2.2 Experimental analysis methods

In this study, an eight-day evaporation experiment was conducted using the shallow-plate evaporation method in the laboratory. Prior to each test, the raw oil samples stored in dark, sealed containers were placed in a constant-temperature water bath to reach room temperature and gently inverted to ensure complete homogenization without creating air bubbles. A precise volume of the homogenized oil was then rapidly transferred onto a pre-weighed shallow evaporating dish using a calibrated syringe to form a continuous oil slick with a controlled uniform thickness of 0.5 mm. An electronic analytical balance (±0.0001 g) was used to measure mass loss over time, simulating the evaporation process of spilled oil under different temperature and wind speed conditions. The specific experimental conditions are summarized in Table 1.

**Table 1:** Experiment conditions

Items	Condition design
Type of test oil	PL, BZ, CFD, SH, CY
Oil film thickness (mm)	0.5
Time (h)	0, 1, 2, 3, 4, 6, 12, 24, 32, 48, 72, 96, 124, 144, 168, 192
Temperature (°C)	7, 15, 20, 25, 30, 35, 37, 40
Wind speed (m s^−1^)	0, 3, 6

The oil distillation process of each test oil was analyzed and determined by gas chromatography according to the ASTM D2887 standard (gasoline, diesel, middle distillate, and heavy oil). The four-component contents (saturates, aromatics, resins, and asphaltenes) were determined using the rod thin-layer chromatography/hydrogen flame ionization detector group component analysis method.

For gas chromatographic analysis of distillation fractions and *n*-alkane distributions (ASTM D2887), approximately 0.1–0.2 g of oil was diluted in an appropriate solvent and injected. For SARA fractionation by TLC-FID, 1–2 µL of diluted oil solution (typically corresponding to 10–20 µg of oil) was spotted on the rods. These are standard analytical-scale quantities used for petroleum characterization.

The density *ρ* of the oil samples at different temperatures was measured in a water bath using the GB/T2540 pycnometer method, and calculated using eqn (1):1
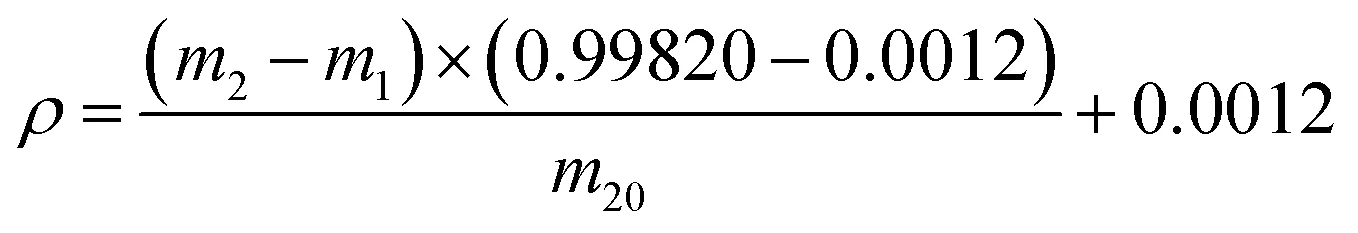
where *m*_1_ is the mass of the empty pycnometer, g; *m*_2_ is the mass of the pycnometer filled with crude oil at temperature *t*, g; *m*_20_ is the mass of the pycnometer filled with water at 20 °C, g.

Kinematic viscosity was measured at different temperatures according to the GB/T 265 petroleum product kinematic viscosity method using calibrated glass capillary viscometers immersed in a temperature-controlled water bath. The time required for a fixed volume of oil to flow through the capillary was recorded with a stopwatch, and the kinematic viscosity as eqn (2):

2 *V*_*t*_ = *C* × *τ*_*t*_

where *C* is the capillary constant, mm^2^ s^−2^; and *τ*_*t*_ is the flow time at temperature *t* °C, s.

The complete experimental workflows, including evaporation, emulsification and physical-property measurement procedures described above, are summarised in Fig. 2.

**Fig. 2:**
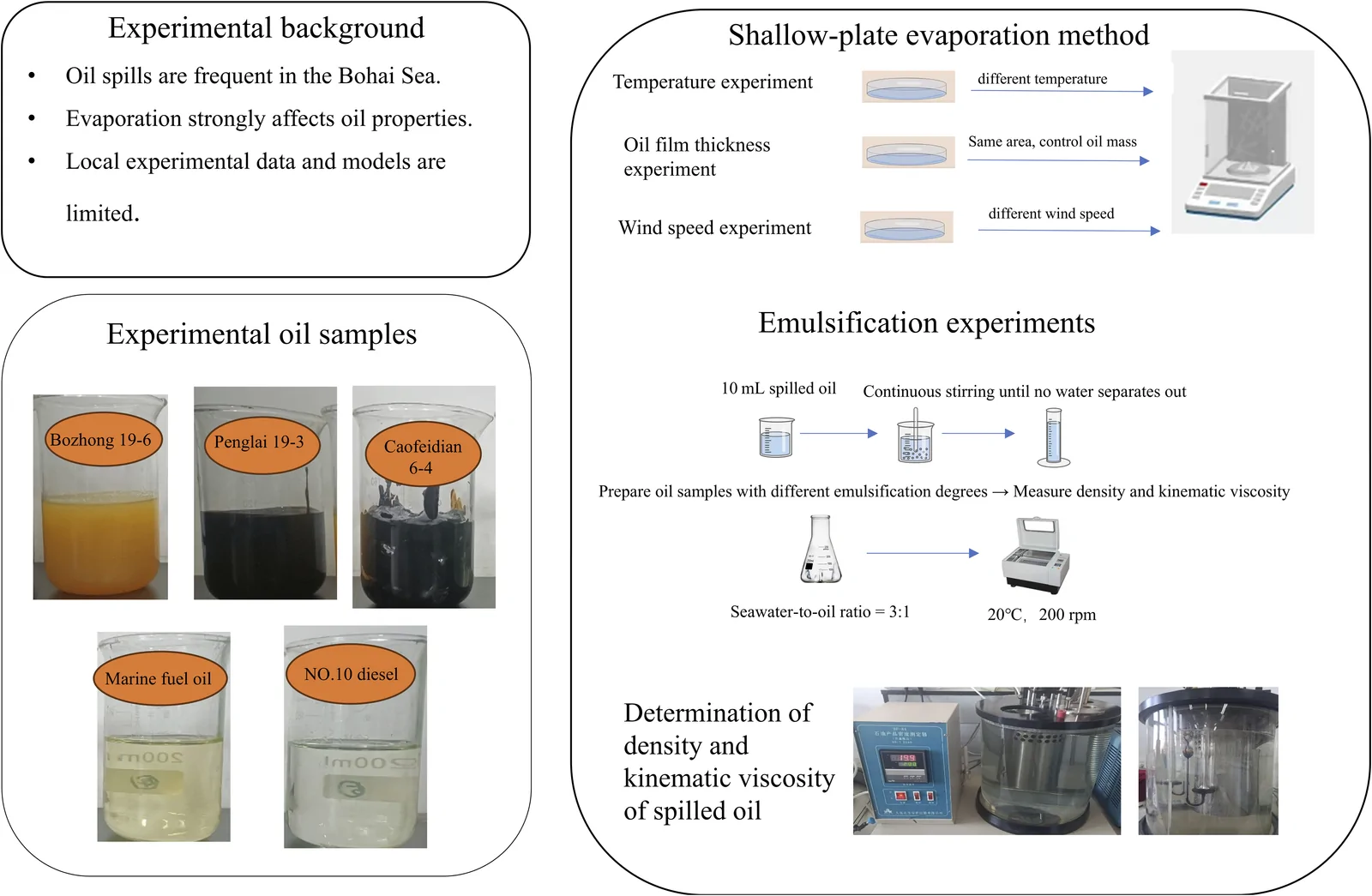
Experimental setup and procedures for oil evaporation, emulsification, and physical property measurements.

## 3 Results and discussion

### 3.1 Basic characteristics of the experimental oils

Petroleum is a complex mixture containing numerous components. Although each type of crude oil has unique molecular and chemical characteristics, advances in analytical chemistry techniques have enabled precise characterization of petroleum components based on their polarity and nonpolarity. Petroleum can be divided into four components: saturates, aromatics, resins, and asphaltenes, and are often the primary data used to characterize oils.^20–22^ In addition, during the distillation process, based on the differences in the boiling points of hydrocarbons, crude oil can be separated into fractions with different boiling ranges, including gasoline, diesel, distillate oil, and heavy oil. Variations in the content of these components significantly affect the physicochemical properties of petroleum. Therefore, component and fraction analysis of petroleum has become an important step in studying its characteristics and property variations. The component and fraction contents of each spilled oil are summarized in Table 2. The distillation fraction data indicate that PL and CFD oils still contain large amounts of heavy fractions at high temperatures, with heavy oil and distillate oil accounting for more than half of the total fractions. In contrast, the other three experimental oils did not exhibit any significant heavy fractions during high-temperature distillation. PL and CFD oils also contain a high proportion of resins and asphaltenes, which is reflected in their physical properties as black, viscous, resinous oils. Conversely, CY and SH oils contain no detectable resins or asphaltenes and exist as transparent liquids.

**Table 2:** Component fractions and basic characteristics of experimental oils

Properties		Oil				
		PL	BZ	CFD	CY	SH
Fraction (%)	Gasoline (G)	6.21	49.10	20.21	9.82	9.98
	Diesel (D)	19.79	33.58	25.72	84.48	83.52
	Distillate oil (F)	36.00	15.87	32.75	5.70	6.50
	Heavy oil (H)	38.00	1.45	21.32	0.00	0.00
Component (%)	Saturates (S)	35.98	94.39	63.05	78.87	77.66
	Aromatics (Ar)	30.32	3.61	17.15	21.13	22.34
	Resins (R)	21.43	1.66	11.59	0.00	0.00
	Asphaltenes (As)	12.27	0.34	8.20	0.00	0.00

During the weathering process (such as evaporation), the chemical composition of spilled oil undergoes significant changes, which strongly affects its physicochemical properties, and further influences other weathering processes, such as diffusion, emulsification, and dissolution. By analyzing the composition of spilled oil before and after weathering using gas chromatography, the weathering process can be preliminarily interpreted and characterized at the molecular level. The figure (Fig. 3) below shows the gas chromatograms of spilled oils before and after 30 days of weathering. The *n*-alkane distributions shown in Fig. 3 were obtained by integrating the peak areas of individual *n*-alkanes (C_10_–C_38_) from the GC chromatograms. Peak areas were normalized to the total identified *n*-alkane area to calculate relative content (%). The bar heights in Fig. 2 directly represent these normalized relative abundances for the original and 30 days weathered oils. Chromatographic analysis reveal that the normal alkanes of light spilled oils such as SH and CY are primarily distributed in the range of C10–C25, whereas those of medium spilled oils such as PL and CFD are distributed over a wider range (C15–C38), containing more high-carbon-number *n*-alkanes, which results in higher density and viscosity. After 30 days of weathering, the C10–C25 alkanes in light spilled oils (such as SH, CY, and BZ) decrease significantly, specifically the low-carbon-number fractions (such as C10–C16), which are nearly completely lost. This finding indicates that weathering has a stronger removal effect on low-carbon-number alkanes. In contrast, medium spilled oils, such as CFD, owing to the higher stability of high-carbon-number alkanes, exhibit low loss (30–50%) and strong resistance to weathering.

**Fig. 3:**
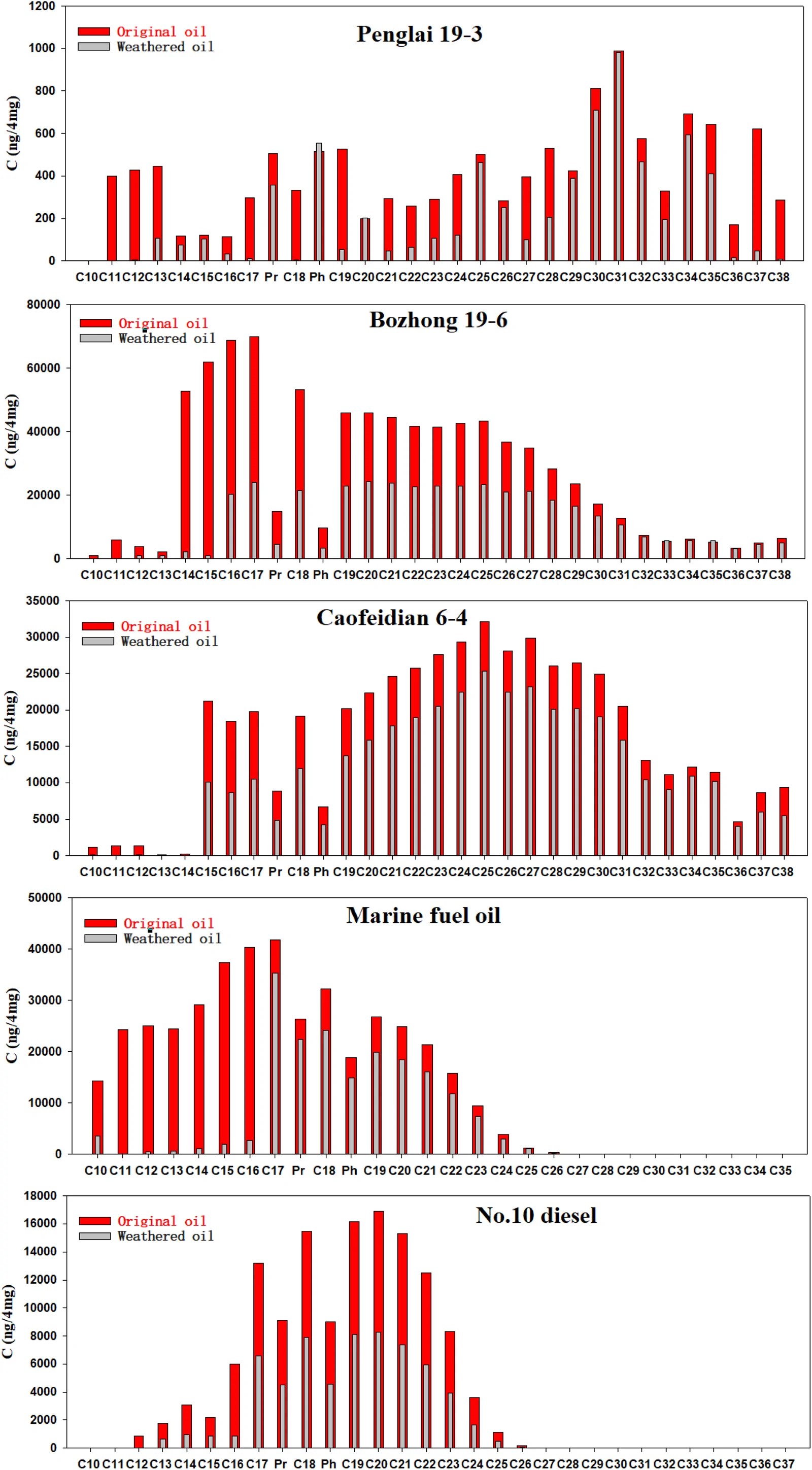
Distribution of *n*-alkanes in spilled oils before and after 30 days of weathering (Penglai 19-3, Bozhong 19-6, Caofeidian 6-4, marine fuel oil, No.10 diesel; horizontal axis: *n*-alkanes; vertical axis: content; legend: original oil, weathered oil).

### 3.2 Analysis of density and kinematic viscosity variation with temperature

Through experimental measurements, the variations in the initial density and kinematic viscosity of each test oil with temperature are shown in Fig. 4. As illustrated in Fig. 4(a), the densities of all test oils decrease with increasing temperature. The higher the temperature, the greater the mass loss of crude oil and the lower its density, with all test oils exhibiting a density reduction of approximately 1.5–3.0%. In the kinematic viscosity measurements, PL and CFD barely flow in the viscometer below 30 °C; therefore, the experimental temperature range for these two oils was set at 30–40 °C. Fig. 4(b) shows that the viscosities of all five test oils decreases with increasing temperature. Among these, the viscosities of CY and SH are less affected by temperature, whereas those of PL and CFD decrease significantly with increasing temperature. In particular, the kinematic viscosity of PL decreases substantially from 1037.36 to 464.34 mm^2^ s^−1^, which may be attributed to its relatively high resin and asphaltene contents.

**Fig. 4:**
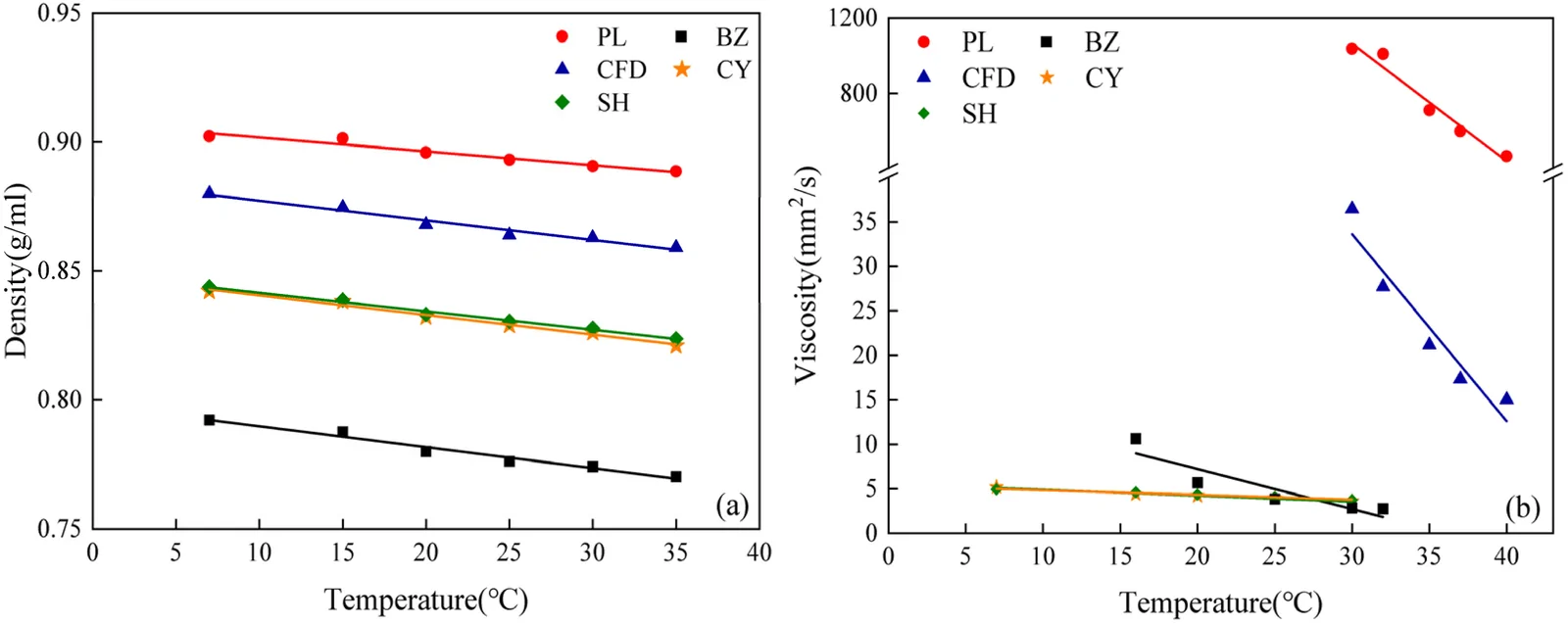
Variations in (a) density and (b) viscosity of test oils at varying temperatures.

Because it is difficult to continuously and timely monitor spilled oil in the marine environment, the effects of different environmental conditions on oil properties can be analyzed and summarized by establishing functional relationships. At present, analyses of the relationships between petroleum density, viscosity, and composition primarily rely on the construction of empirical formulas and fitted functions.^23^ The component fractions of spilled oils, serving as a unified dataset distinguishing different oils, can be applied in constructing functional relationships for oil properties. By correlating the equation parameters with oil sample components and fraction data, a generalized empirical equation of oil group composition–property can be established. Based on the data of the five test oils under different temperatures, as shown in Fig. 3, it is observed that density and viscosity exhibit linear relationships with temperature.

For density *ρ*, parameters *a*_1_ and *β*_1_ were set, and linear fitting was performed for each oil group (Table 3).

**Table 3:** Fitting results of density and temperature for test oils

Test oil	*ρ* = *α*_1_ × *T* + *β*_1_	*R* ^2^
PL	*ρ* = −5.38 × 10^−4^ × *T* + 0.91	0.9394
BZ	*ρ* = −8.13 × 10^−4^ × *T* + 0.80	0.9663
CFD	*ρ* = −7.57 × 10^−4^ × *T* + 0.88	0.9613
CY	*ρ* = −7.62 × 10^−4^ × *T* + 0.85	0.9829
SH	*ρ* = −7.17 × 10^−4^ × *T* + 0.85	0.9883

By fitting, the slope (*α*_1_) and intercept (*β*_1_) of the density equation parameters for each oil were obtained. These parameters differ considerably among the oil groups because of their distinct bulk compositions. Oils richer in saturates (*e.g.*, BZ) exhibit steeper density–temperature slopes, reflecting greater thermal expansion, whereas oils richer in resins and asphaltenes (*e.g.*, PL and CFD) display higher intercepts, consistent with denser molecular packing. These composition-driven differences provide the physicochemical basis for the observed parameter variations and are essential for developing generalized predictive models. The fraction content of oil components can serve as a unified variable to distinguish different oils. Therefore, the parameters of each oil equation were correlated with the corresponding component fractions, resulting in an empirical equation of density variation expressed by the oil components (Table 4). In the fitting results, *α*_1_ and *β*_1_ are closely related to the saturated fraction content (*R*^2^ = 0.8423 and 0.9957, respectively). The large variation in *R*^2^ values across different components indicates that saturates are the dominant compositional driver of density variation with temperature for the studied Bohai oils, while components with weaker correlations play secondary roles under the experimental conditions (eqn (3) and (4)):

3 *a*_1_ × 10^6^ = −4.42 × *S* − 408.41

4 *β*_1_ = −0.0063 × e^(0.032×*S*)^ + 0.93

where *S* is the saturated fraction content of the test oil (%).

**Table 4:** Fitting results of *α*_1_, *β*_1_ with oil components and determination coefficients *R*^2^

Component	*α*_1_	*R* ^2^	*β*_1_	*R* ^2^
G	*α*_1_ × 10^6^ = −3.89*G* − 643.34	0.4174	*β*_1_ = −0.0018*G* + 0.89	0.5814
S	*α*_1_ × 10^6^ = −4.42*S* − 408.41	0.8423	*β*_1_ = −0.0063 × e^(0.032×*S*)^ + 0.93	0.9957
D	*α*_1_ × 10^6^ = −1.09*D* − 663.91	0.1071	*β*_1_ = −4.41*D* + 0.88	0.1146
R	*α*_1_ × 10^6^ = 9.08*R* − 780.53	0.6538	*β*_1_ = 0.0036*R* + 0.83	0.6719
F	*α*_1_ × 10^6^ = 4.15*F* − 797.84	0.3143	*β*_1_ = 0.002*F* + 0.82	0.4732
As	*α*_1_ × 10^6^ = −2.31As − 707.93	0.0156	*β*_1_ = −0.0013As + 0.86	0.0296

By substituting the above expressions (eqn (3) and (4)) for the linear equation parameters *a*_1_ and *β*_1_, an empirical equation (eqn (5)) of the initial density of spilled oils with different saturated fraction contents varying with temperature can be obtained.5
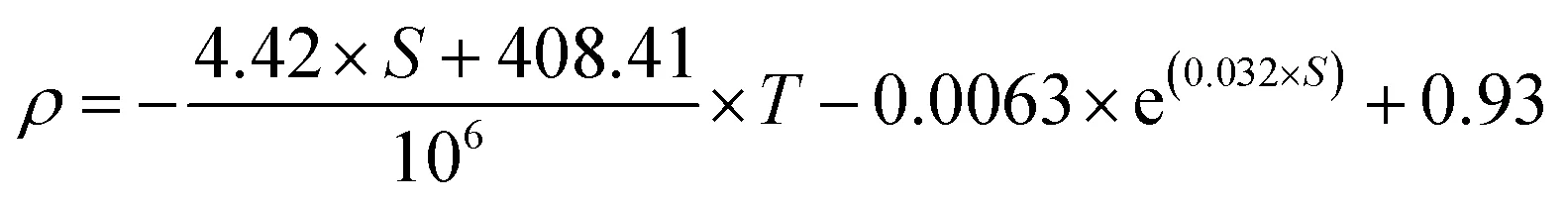


Similarly, for kinematic viscosity, parameters *a*_2_ and *β*_2_ are set, and linear fitting is performed on the viscosity *V*_*t*_ of the test oils with temperature *T*. The results are summarized in Table 5:

**Table 5:** Fitting results of viscosity and temperature for test oils

Test oil	*V*_*t*_ = *a*_2_ × *T* + *β*_2_	*R* ^2^
PL	*V*_*t*_ = −62.64 × *T* + 2943.51	0.9534
BZ	*V*_*t*_ = −0.38 × *T* + 14.71	0.6933
CFD	*V*_*t*_ = −2.1 × *T* + 96.65	0.8986
CY	*V*_*t*_ = −0.067 × *T* + 5.58	0.9784
SH	*V*_*t*_ = −0.053 × *T* + 5.38	0.9707

Following the same fitting process as with the density equation parameters, the viscosity equation parameters of each oil group are correlated with the component fractions (Table 6). Resin content (*R*) exhibits the strongest correlations because resins are the primary contributors to high viscosity in petroleum due to their large molecular size, polarity, and ability to form intermolecular associations. The following empirical expressions are therefore obtained (eqn (6) and (7)):where *R* is the resin content (%):

6 *a*_2_ = 0.034 × e^(0.35×*R*)^ + 0.12

7 Ln(*β*_2_) = 0.35 × *R* + 0.57

where *R* is the resin content (%).

**Table 6:** Fitting results of *α*_2_, *β*_2_ with oil components and determination coefficients *R*^2^

Component	*α*_2_	*R* ^2^	*β*_2_	*R* ^2^
G	*α*_2_ = 0.64 × *G* − 25.26	0.1650	Ln(*β*_2_) = −1.71*G* + 18.6	0.9986
S	*α*_2_ = −5981.33 × e^(−0.13×*G*)^ + 0.015	0.9999	Ln(*β*_2_) = −0.13*S* + 12.57	0.9999
D	*α*_2_ = −6.64 × 10^6^ × e^(−0.58×*D*)^ − 0.16	0.9999	Ln(*β*_2_) = −0.58*D* + 19.38	0.9999
R	*α*_2_ = −0.034 × e^(0.35×*R*)^ − 0.12	0.9999	Ln(*β*_2_) = 0.35*R* + 0.57	0.9999
F	*α*_2_ = −1.3*F* + 12.15	0.4513	Ln(*β*_2_) = 1.05*F* − 29.85	0.9999
As	*α*_2_ = −0.0018 × e^(0.85×As)^ − 0.16	0.9999	Ln(*β*_2_) = 0.84As − 2.31	0.9999

Based on the above parameter expressions, an integrated empirical equation (eqn (8)) of spilled oil viscosity variation with temperature under different resin contents is obtained.

8 *V*_*t*_ = [0.034 × e^(0.35×*R*)^ + 0.12] × *T* + e^(0.35×*R*+0.57)^

### 3.3 Evaporation kinetics of oil spills

The shallow-pan evaporation experiment is one of the most commonly used method for studying oil spill evaporation. It is applied to investigate the effects of factors such as wind speed, temperature, and different oil samples on the evaporation process, and further to analyze the evaporation kinetics and rate equations under varying conditions.^10^ In this study, evaporation dishes containing a fixed quantity of different test oils were placed under various temperature and wind speed conditions for eight days. At different evaporation time points, the dishes were weighed sequentially, and the evaporation loss rate was calculated. The evaporation processes of the five test oils under different temperatures and wind speeds are shown in Fig. 5. As shown in Fig. 5(a)–(e), under different temperature conditions, the evaporation rates of the five test oils increase rapidly within the first 20 h, then gradually decrease after 60 h, and finally approach asymptotic maximum values. This plateau occurs because evaporation is selective: low-boiling-point components (mainly C_10_–C_16_*n*-alkanes and light aromatics) are lost preferentially in the early stage. Once these volatile fractions are depleted, the residual oil is dominated by high-boiling-point saturates, resins and asphaltenes that have extremely low vapor pressures at 7–40 °C. Consequently, further mass loss becomes negligible and the evaporation rate stabilizes. Among the five test oils, PL exhibits the lowest evaporation rate, increasing from 9.7% to 13.1%. The evaporation behavior of CFD follows a similar trend to that of PL, with its maximum evaporation rate slightly higher than that of PL. For the three light oils, BZ, CY, and SH, the maximum evaporation rates are generally between 30% and 70%. The large differences in evaporation extent among the five oils can be directly related to their molecular composition. As shown in Fig. 3 and Table 2, the light oils (BZ, CY and SH) are dominated by low-to-middle carbon number *n*-alkanes (mainly C_10_–C_25_) and contain almost no resins or asphaltenes. These light molecules have high vapor pressure and are preferentially lost, resulting in maximum evaporative losses of 30–70%. In contrast, PL and CFD contain substantial amounts of high-carbon *n*-alkanes (up to C_38_) together with high contents of resins and asphaltenes. These heavy molecular fractions have very low volatility under the experimental temperatures, which explains their limited maximum evaporation (only 9–15%). Thus the observed evaporation kinetics are controlled by the relative abundance of these specific chemical groups rather than by the bulk oil type alone. This result is consistent with Fingas's finding,^24^ which states that light crude oils evaporate most of their components as temperature increases, whereas nonlight oils evaporate much less. Higher temperatures significantly increase the evaporation and maximum evaporation rates of oil spills. When the temperature increases from 7 to 35 °C, the maximum evaporation rates of all test oils increase by approximately 50%. For nonlight oils, the promoting effect of temperature on evaporation is lower. Fingas^24^ suggested that the more volatile components petroleum contains, the greater its evaporation extent and rate, whereas medium crude oils contain several components that barely evaporate even after prolonged exposure to high temperatures.

**Fig. 5:**
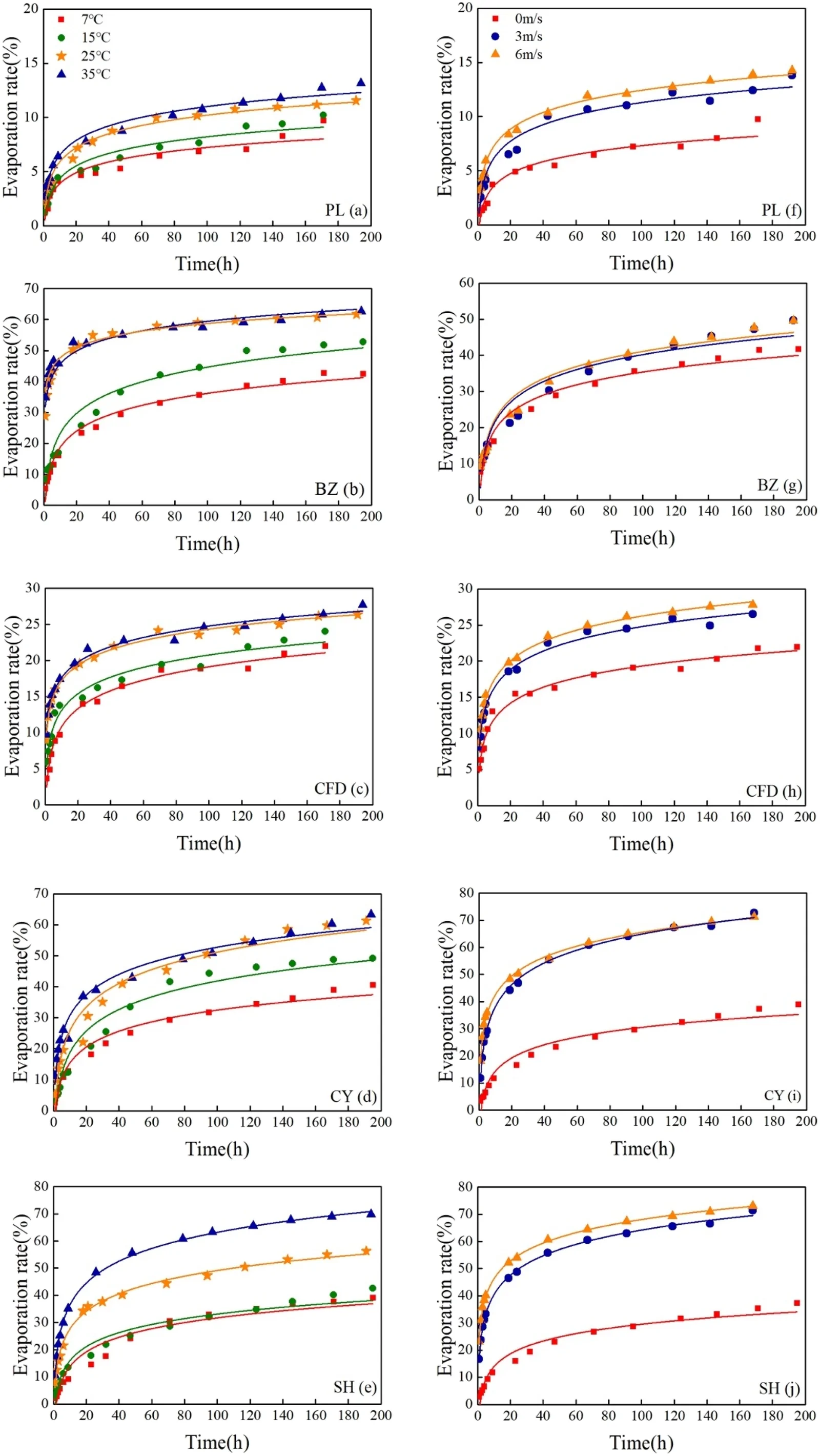
Evaporation kinetics of test oils; (a)–(e) evaporation loss under different temperatures; (f)–(j) evaporation loss under different wind speeds.

Wind speed exhibits a similar promoting effect on evaporation as temperature. As shown in Fig. 5(f)–(j), with increase in time, the evaporation rate of oil spills initially increases rapidly and then decreases. Under windy conditions, the evaporation rate and maximum evaporation rate are significantly higher than that under calm conditions. An increase in the wind speed from 3 to 6 m s^−1^ promotes evaporation, though not markedly, which is consistent with the findings of Fingas.^20^ The evaporation of BZ oil is less affected by temperature, and with increasing wind speed, its maximum evaporation rate increases by only approximately 8%.

In studies on the evaporation process of petroleum, researchers often summarize empirical equations based on experimental results, revealing that all naturally occurring hydrocarbon mixtures (such as crude oil and petroleum products) generally follow logarithmic or exponential evaporation curves.^25^ From these evaporation kinetics plots, it can be observed that the evaporation rate *E* of each oil spill follows the evaporation equation, exhibiting logarithmic relationship with evaporation time *t*, expressed as ln(*t*). Therefore, the evaporation data of each test oil can first be fitted according to eqn (9) to obtain the evaporation rate equations under different conditions.

9 *E* = *α* × ln(*t*) + *β*

After obtaining the evaporation rate equations of the test oils (Table 7), notably, the different oils exhibited distinct evaporation equations under different conditions. To obtain an empirical expression that can uniformly express the evaporation rate of each test oil under different conditions, a method is required to uniformly and accurately predict evaporation using other data.^26^ Initially, the influence coefficients of temperature and wind speed were set, and their influence on the slope *α* and intercept *β* of the evaporation equation was analyzed through linear fitting, and then a unified expression between wind speed and evaporation equation parameters was established. Next, the influence coefficients in this expression were correlated with the component data of the test oils, and significant factors were selected based on the fitting correlation coefficients. Finally, an empirical evaporation rate equation was constructed, incorporating oil composition, temperature, and time variables. The fitting results of the evaporation kinetics equation for the temperature part evaporation rate *E* in the logarithmic form of evaporation time ln(*t*) are summarized in Table 7.

**Table 7:** Evaporation rate equations of test oils under different temperatures

Test oil	Temperature			
	7 °C	15 °C	25 °C	35 °C
PL	*E* = 1.4566 ln *τ* + 0.5241	*E* = 1.6732 ln *τ* + 0.5082	*E* = 1.8778 ln *τ* + 1.5636	*E* = 1.9507 ln *τ* + 2.0245
	*R* ^2^ = 0.9338	*R* ^2^ = 0.9575	*R* ^2^ = 0.9821	*R* ^2^ = 0.9821
BZ	*E* = 7.5853 ln *τ* + 1.2384	*E* = 9.3218 ln *τ* + 1.7269	*E* = 5.9923 ln *τ* + 31.851	*E* = 4.8332 ln *τ* + 36.487
	*R* ^2^ = 0.9824	*R* ^2^ = 0.9601	*R* ^2^ = 0.9797	*R* ^2^ = 0.9837
CFD	*E* = 3.6187 ln *τ* + 2.4611	*E* = 3.3469 ln *τ* + 5.3451	*E* = 3.0395 ln *τ* + 10.289	*E* = 3.1346 ln *τ* + 10.29
	*R* ^2^ = 0.9827	*R* ^2^ = 0.9744	*R* ^2^ = 0.9785	*R* ^2^ = 0.9918
CY	*E* = 7.4228 ln *τ* − 1.7252	*E* = 10.036 ln *τ* − 4.3715	*E* = 11.018 ln *τ* + 0.4138	*E* = 9.592 ln *τ* + 8.5824
	*R* ^2^ = 0.9741	*R* ^2^ = 0.9578	*R* ^2^ = 0.9651	*R* ^2^ = 0.9784
SH	*E* = 7.807 ln *τ* − 4.2683	*E* = 7.4728 ln *τ* − 1.3356	*E* = 9.3728 ln *τ* + 5.9729	*E* = 11.629 ln *τ* + 9.6322
	*R* ^2^ = 0.9482	*R* ^2^ = 0.9618	*R* ^2^ = 0.9954	*R* ^2^ = 0.9992

From the above table, it can be inferred that the evaporation rate equations of each oil group differ under different temperatures. To ultimately establish a unified empirical evaporation equation, the slopes (*α*) and intercepts (*β*) of the evaporation rate equations at different temperatures were separately fitted and analyzed.

Initially, the slope *α* of each test oil equation was correlated with temperature *T*, and influence coefficients *a*_3_ and *β*_3_ were introduced for parameterization. Based on this, an *α*–*T* relationship was constructed containing parameters *a*_3_ and *β*_3_. To further improve the generality of the model, the same analytical approach used for oil spill characteristic analysis was adopted, linking *a*_3_ and *β*_3_ with oil composition characteristics. The results showed that *a*_3_ was primarily influenced by gasoline fraction content, whereas *β*_3_ exhibited a significant correlation with saturate content. On this basis, we established empirical equations containing oil composition parameters (eqn (10) and (11)), providing a unified computational framework for predicting the evaporation behavior of oils with different properties.

10 *a*_3_ × 10^3^ = −4.94 × *G* + 112.43

11 *β*_3_ = 0.14 × *S* − 4.33

where *G* is the gasoline fraction content (%) and *S* is the saturate content (%).

By combining the empirical equations (eqn (10) and (11)), including oil composition fractions, with the linear equation of slope *α*, an empirical expression (eqn (12)) was obtained to describe the variation of slope *α* with gasoline fraction, saturate content, and temperature.12
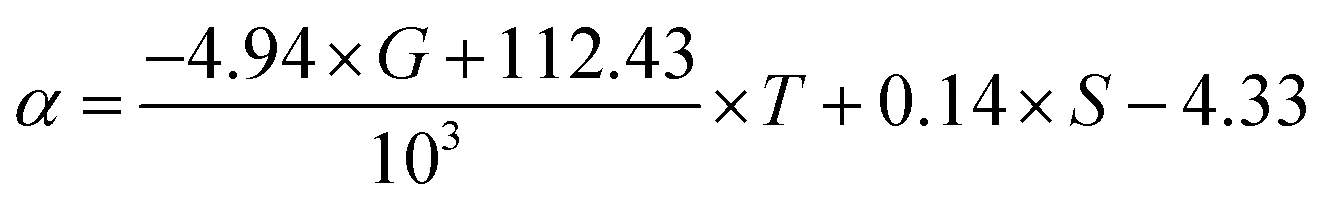


After expressing the slope *α* in terms of composition and fraction data, the intercept *β* of the oil evaporation equation was analyzed similarly. Influence coefficients *a*_4_ and *β*_4_ were introduced and linearly fitted with temperature *T*. By correlating *a*_4_ and *β*_4_ with oil composition, and selecting the results with the highest correlation, we obtained unified empirical equations (eqn (13) and (14)).

13 Ln(*a*_4_) = 0.066 × *S* − 5.82

14 *β*_4_ = 0.87 × As − 8.96

where *S* is the saturate content (%) and As is the asphaltene content (%).

Finally, the empirical expression for the intercept *β* of the evaporation equation was obtained (eqn (15)).

15 *β* = e^(0.066×*G*−5.82)^ × *T* + 0.87 × As − 8.96

Through the above fitting process, the empirical expressions for the slope *α* and intercept *β* of the evaporation rate equation (eqn (12) and (15)) were derived. By substituting these into the evaporation equation (eqn (9)), a unified empirical equation (eqn (16)), describing the evaporation rate of oil spills over time as a function of saturate content, gasoline fraction, asphaltene content, and temperature, was obtained.16
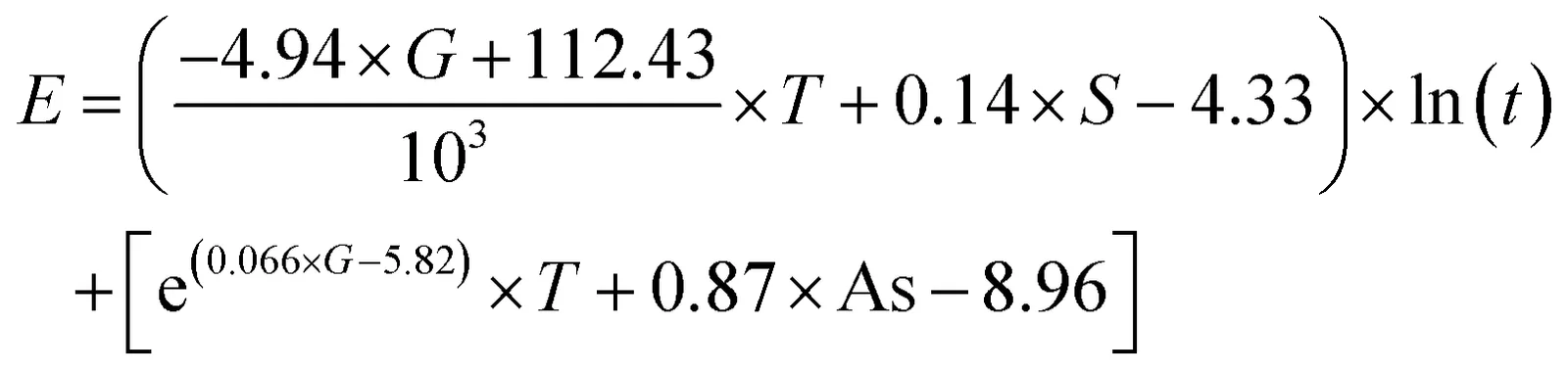


Wind speed is another important factor that influences the evaporation process of oil spills and its effects on the oil spill evaporation process also needs to be analyzed. Following the same procedure as for temperature, the evaporation rate of each oil under different wind speed conditions was linearly fitted according to eqn (9). The evaporation rate equations of each test oil at different wind speeds are summarized in Table 8.

**Table 8:** Evaporation rate equations of test oils under different wind speeds

Test oil	Wind speed		
	0 m s^−1^	3 m s^−1^	6 m s^−1^
PL	*E* = 1.5498 ln *τ* + 0.1485	*E* = 2.2224 ln *τ* + 1.0541	*E* = 2.2322 ln *τ* + 2.1438
	*R* ^2^ = 0.926	*R* ^2^ = 0.9667	*R* ^2^ = 0.9893
BZ	*E* = 6.8708 ln *τ* + 3.7422	*E* = 8.016 ln *τ* + 3.2824	*E* = 8.0828 ln *τ* + 3.8536
	*R* ^2^ = 0.9758	*R* ^2^ = 0.9505	*R* ^2^ = 0.9654
CFD	*E* = 3.2038 ln *τ* + 4.5611	*E* = 3.6993 ln *τ* + 7.7396	*E* = 3.849 ln *τ* + 8.5035
	*R* ^2^ = 0.9848	*R* ^2^ = 0.9914	*R* ^2^ = 0.9973
CY	*E* = 7.0497 ln *τ* − 1.9131	*E* = 11.665 ln *τ* + 11.236	*E* = 10.032 ln *τ* + 19.382
	*R* ^2^ = 0.962	*R* ^2^ = 0.9979	*R* ^2^ = 0.9979
SH	*E* = 6.7813 ln *τ* − 1.7206	*E* = 10.32 ln *τ* + 16.655	*E* = 9.4682 ln *τ* + 24.462
	*R* ^2^ = 0.9683	*R* ^2^ = 0.9983	*R* ^2^ = 0.9987

Initially, the evaporation equation slope *α* was fitted against wind speed *V*. During the fitting process, *α* and *V* did not show an evident linear relationship, but rather followed a square-root form of wind speed. Therefore, influence coefficients *a*_5_ and *β*_5_ were introduced, and a linear fit was performed in the form of *V*^1/2^. By correlating *a*_5_ and *β*_5_ with oil fractions, empirical equations (eqn (17) and (18)) were obtained.

17 *a*_5_ = 0.14 × e^[29.43/(*F*+6.81)]^

18 *β*_5_ = −0.47 × As + 7.21

where *F* is the fraction oil content (%) and As is the asphaltene content (%).

Therefore, the expression of slope *α* under different wind speeds is as follows (eqn (19)):

19 *α* = 0.14 × e^[29.43/(*F*+6.81)]^ × *V*^1/2^ − 0.47 × As + 7.21

For the intercept *β* of evaporation equation against wind speed *V*, influence coefficients *a*_6_ and *β*_6_ were introduced for linear fitting. Finally, by correlating *a*_6_ and *β*_6_ with the oil fractions of the test oils eqn (20) and (21) were obtained.

20 *a*_6_ = 0.061 × *D* − 1.21

21 *β*_6_ = −10.82 × e^(−0.11×*G*)^ + 4.36

where *D* is the diesel fraction content (%) and *G* is the gasoline fraction content (%).

The final expression for parameter *β* of the evaporation equation was obtained (eqn (22)).

22 *β* = (0.061 × *D* − 1.21) × *V* − 10.82 × e^(−0.11×*G*)^ + 4.36

By combining the empirical expressions for the evaporation equation parameters (eqn (19) and (22)), the empirical equation describing the evaporation rate as a function of oil fractions, wind speed, and time were finally derived (eqn (23)).23
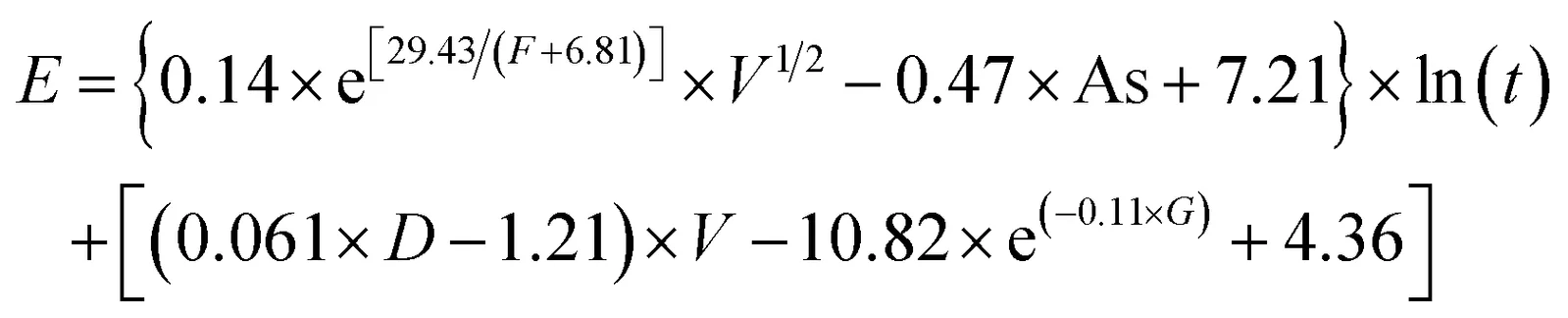


### 3.4 Effect of evaporation process on density and kinematic viscosity of oil spills

Based on the previous analysis of evaporation rates under different environmental conditions, this section further analyzes the influence of evaporation on oil spill characteristics and develops empirical formulas to calculate the changes in oil properties. The properties of each test oil at different evaporation rates were measured and analyzed, with oil samples prepared at different evaporation rates according to the corresponding evaporation durations from the earlier evaporation experiments. The variations of density and viscosity under different evaporation rates for different oil groups are shown in Fig. 6.

**Fig. 6:**
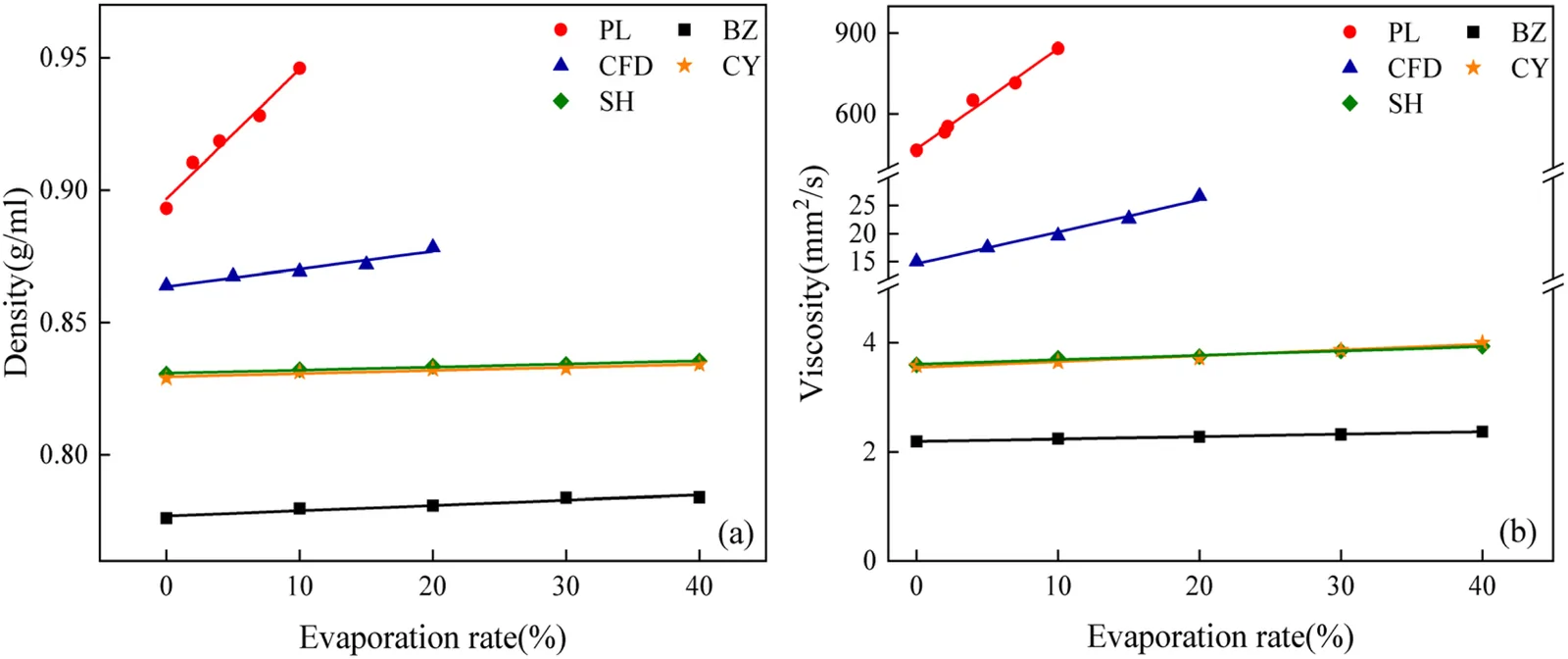
Density (a) and viscosity (b) variations of each test oil at different evaporation rates.

As shown in Fig. 6(a) and (b), density and kinematic viscosity of the oil spill increase with evaporation rate in the early and middle stages. However, both properties approach limiting values once the evaporation rate itself plateaus. This is because, after the preferential loss of light components, the residual oil consists mainly of high-boiling-point heavy fractions whose further evaporative loss and density change become negligible under the experimental conditions. These variations are attributed to a higher evaporation rate, which leads to an increase in the relative concentration of heavy molecules. Consequently, the density and viscosity of the floating oil increases.^27^ However, because the oils BZ, CY, and SH contain nearly negligible heavy components (resins and asphaltenes ≈ 0%), the evaporation process preferentially removes low-molecular-weight saturates and aromatics, resulting in only minor relative enrichment of residual heavy molecules. Consequently, the increase in kinematic viscosity remains limited. In contrast, PL and CFD, which contain substantial resins and asphaltenes, experience a marked rise in the relative concentration of these high-viscosity constituents upon loss of volatiles, leading to a pronounced viscosity increase. This compositional difference explains why resin content is the key factor determining the extent to which evaporation influences viscosity. This result is consistent with the earlier analysis of oil properties under different temperatures (eqn (10)), which indicates that resin content is the key factor determining the extent to which evaporation influences viscosity. The higher the resin content, the greater the effect of evaporation on oil spill viscosity. In the previous analysis of density and viscosity variations with temperature, the properties of the initial oil samples were fitted to obtain eqn (5) and (8), which apply only to the density and viscosity at the initial stage of oil spills. To further explore the effect of evaporation on oil spill properties, the methodology consistent with the earlier oil characteristic analysis was adopted, combining evaporation-rate analysis with oil property analysis. Next, empirical equations were derived to describe the variation of oil density and viscosity at different evaporation stages, providing quantitative tools for a deeper understanding of oil spill behavior under evaporation.

For the density analysis, influence coefficients *a*_7_ and *β*_7_ were introduced, and an empirical expression for oil density as a function of evaporation rate *E* was obtained through linear fitting (Table 9). The empirical expression of evaporation rate *E* has been derived in the previous section on oil spill evaporation rate analysis. By correlating the influence coefficients *a*_7_ and *β*_7_, obtained from the fitting results of each oil group in Table 9, with the component fractions (Table 10), eqn (24) and (25) were obtained.

24 Ln(*a*_7_) = 0.19 × *R* − 9.44

25 *β*_7_ = −0.002 × *S* + 0.98

where *R* is resin content (%) and *S* is saturate content (%).

**Table 9:** Fitting results of test oil density with evaporation rate

Test oil	*ρ* = *a*_7_ × *E* + *β*_7_	*R* ^2^
PL	*ρ* = 4.91 × 10^−3^ × *E* + 0.9	0.9737
BZ	*ρ* = 1.97 × 10^−4^ × *E* + 0.78	0.9285
CFD	*ρ* = 6.66 × 10^−4^ × *E* + 0.86	0.9446
CY	*ρ* = 1.19 × 10^−4^ × *E* + 0.83	0.9346
SH	*ρ* = 1.17 × 10^−4^ × *E* + 0.83	0.9769

**Table 10:** Fitting results of *α*_7_, *β*_7_ with oil components and determination coefficient *R*^2^

Component	*α*_7_	*R* ^2^	*β*_7_	*R* ^2^
G	Ln(*α*_7_) = −1.06 × *G* + 1.25	0.9695	*β*_7_ = −0.002*G* + 0.88	0.5934
S	Ln(*α*_7_) = −0.076 × *S* − 2.59	0.9974	*β*_7_ = −0.002*S* + 0.98	0.9362
D	Ln(*α*_7_) = −0.32 × *D* + 1.05	0.9976	*β*_7_ = 4.47 × e^(−0.2×*D*)^ + 0.82	0.6407
R	Ln(*α*_7_) = 0.19 × *R* − 9.44	0.9994	*β*_7_ = 0.0039*R* + 0.81	0.6954
F	Ln(*α*_7_) = 0.6*F* − 26.87	0.9954	*β*_7_ = 0.0021*F* + 0.8	0.4659
As	Ln(*α*_7_) = 0.47 × As − 11.08	0.9972	*β*_7_ = 0.0067As + 0.81	0.7399

Based on the parameter relationships provided in eqn (16), (24) and (25), they were substituted into the original linear equation, and the empirical equation (eqn (26)) describing the density variation of oil spills with different component fractions as a function of temperature and time was finally obtained.

26 *ρ* = e^(0.19×*R*−9.44)^ × *E* − 0.002 × *S* + 0.98

Similarly, by setting influence coefficients *a*_8_ and *β*_8_, a fitting analysis of oil viscosity was performed (Table 11).

**Table 11:** Fitting results of test oil viscosity with temperature

Test oil	*V*_*t*_ = *a*_8_ × *E* + *β*_8_	*R* ^2^
PL	*V*_*t*_ = 37.35 × *E* + 469.63	0.9839
BZ	*V*_*t*_ = 0.0044 × *E* + 2.19	0.9954
CFD	*V*_*t*_ = 0.57 × *E* + 14.62	0.9842
CY	*V*_*t*_ = 0.011 × *E* + 3.55	0.9712
SH	*V*_*t*_ = 0.0082 × *E* + 3.61	0.9779

To obtain further correlation with oil component fractions (Table 12), empirical equations (eqn (27) and (28)) were obtained for *a*_8_ and *β*_8_ with components.

27 Ln(*a*_8_) = 0.43 × *R* − 5.49

28 Ln(*β*_8_) = −0.13 × *S* + 10.7

where *R* is resin content (%) and *S* is saturate content (%).

**Table 12:** Fitting results of *α*_8_, *β*_8_ with oil components and determination coefficient *R*^2^

Component	*α*_8_	*R* ^2^	*β*_8_	*R* ^2^
G	Ln(*α*_8_) = −3.45 × *G* + 25.02	0.9997	Ln(*β*_8_) = −1.33*G* + 14.39	0.9987
S	Ln(*α*_8_) = −0.16 × *S* + 9.22	1	Ln(*β*_8_) = −0.13*S* + 10.7	0.9999
D	Ln(*α*_8_) = −0.71 × *D* + 17.58	1	Ln(*β*_8_) = −0.58*D* + 17.72	0.9998
R	Ln(*α*_8_) = 0.43 × *R* − 5.49	1	Ln(*β*_8_) = 0.35*R* − 1.35	0.9999
F	Ln(*α*_8_) = 1.29*F* − 42.72	1	Ln(*β*_8_) = 1.07*F* − 32.28	0.9998
As	Ln(*α*_8_) = 1.03 × As − 8.99	1	Ln(*β*_8_) = 0.85As − 4.3	0.9998

By integrating eqn (16), (27) and (28) into the original linear equation, the empirical equation (eqn (29)) describing the kinematic viscosity of oil spills with different component fractions as a function of temperature and time is obtained.

29 *V*_*t*_ = e^(0.43×*R*−5.49)^ × *E* + e^(−0.13×*S*+10.7)^

## 4 Conclusion

The behavioral characteristics of oil spills in marine environments are primarily influenced by temperature and wind speed. Using the shallow plate evaporation experimental method, this study simulated the evaporation processes of five test oils under varying environmental conditions and systematically analyzed the effect of evaporation on the physicochemical properties of oil spills. The results provide valuable data for predicting the behavior of marine oil spills and establish corresponding empirical calculation models to improve the simulation and forecasting capability of oil spill migration and transformation processes.

(1) Through experiments, the variation patterns of the characteristic parameters of each test oil with temperature were obtained. Density and kinematic viscosity decreased gradually with increasing temperature. Based on the fitting analysis of the experimental data, empirical equations describing the variation of initial physicochemical properties of different oil spills were established. These models can be applied to predict the initial behavior of oil spills in real marine environments.

(2) Kinetic analysis of the evaporation process showed that increase in temperature and wind speed promote oil spill evaporation. Under different temperature and wind speed conditions, all oils exhibited an initial trend of rapid increase followed by stabilization. The evaporation rate equations were consistent with the logarithmic form *α* × ln(*t*) + *β*. By correlating the evaporation rate equation parameters with oil composition, empirical equations of oil evaporation rates under different temperature and wind speed conditions were obtained, enabling the prediction of evaporation losses based on oil composition and distillation fractions.

(3) As evaporation progressed, the density and viscosity of oil spills increased, particularly for PL and CFD. By fitting these changes with oil composition and distillation fractions, empirical equations describing the variation of density and kinematic viscosity during different evaporation stages were derived. These equations can be used to predict the effects of evaporation on oil spill properties, supporting the development of relevant emergency response strategies.

Overall, the empirical models developed in this study enhance the quantitative understanding of oil-spill evaporation and property evolution under varying environmental conditions. Future work will focus on integrating these laboratory-derived equations into numerical oil-spill trajectory models and validating them under real marine field conditions in the Bohai Sea.

## Author contributions

LW and XG conceived and designed the research. LW conducted the data analysis and wrote the original draft. All authors contributed to writing, reviewing, editing and approved the manuscript for publication.

## Conflicts of interest

The authors have no competing interests to declare that are relevant to the content of this article.

## Data Availability

The data presented in this study are available on reasonable request from the corresponding author.
